# Notes on Preparations for the Sick

**Published:** 1889-03

**Authors:** 


### Notes on Preparations for the Sick.

**Prof. Dr. Kemmerich's Preparations of Beef.**—F. W. Berk & Co., London.

We have received samples of these excellent preparations. Chemical analysis shows that they are of the highest nutritive value, and are superior to other well-known meat-extracts.

The **Extract of Beef** is richer in nitrogen than any other similar extract: it contains 7.86 per cent. of soluble albumen, and 18.24 of salts, chiefly phosphates with potassium.

The **Peptone of Beef**, according to an analysis by Dr. Clark, of Glasgow, contains 35.10 per cent. of peptone, with 11.25 of soluble albuminoids. It is readily soluble in hot water, and the solution has an agreeable flavour. The nutritive value of peptones, and the fact that they require no digestion, are too well known to require comment: all authorities agree as to the large proportion of nutritive matter in these preparations, which are manufactured from the best of raw material at Santa Elena, in the Argentine Republic. They are both of the highest value in the treatment of the sick, not simply as occasional stimulants and restoratives, but because they supply good nitrogenous pabulum for the renovation of nitrogenous tissues, and in a most agreeable form which any invalid can take, even when other food cannot be swallowed or digested. The beef peptone is also very suitable for administration by enema when rectal feeding is desirable or necessary.

---

**Soden Mineral Pastilles.**—The Soden Mineral Produce Company, London.

The value of the medicinal springs of Soden, in the Taunus, has long been recognised: these pastilles provide the entire benefit of the Taunus waters to those who cannot afford a long journey and an expensive sojourn in foreign hotels. They are found to be of value in catarrhal affections of the throat and air-passages, and are much appreciated by those patients who have given them a trial.

**Tabloid Triturates.**—BURROUGHS, WELLCOME & Co., London.

These tabloid triturates are creating a demand, founded upon the following advantages:—

1. Absolute purity and uniformity of the drug.
2. Absolute accuracy of dosage.
3. They offer the most pleasing and palatable form for the administration of the nauseous drugs usually prescribed in the form of tinctures or powders.

We have found them of much service to patients who cannot be induced to swallow any medicine in form of pill or mixture, and to those who require frequent doses, which can be carried in the pocket and easily swallowed.

---

**Sozoiodol-Salts.**—FREDERICK BOEHM, London.

We have received samples of some of these, viz.:—

Potassium Sozoiodol.
Sodium ,,
Zinc ,,
Mercury ,,

Sozoiodol—which is spoken of as "an odourless substitute for iodoform"—is a compound of three powerful antiseptics, in the following proportions:—

Iodine, 52 to 54 per cent.
Carbolic Acid, 20 per cent.
Sulphur, 7 per cent.

It is itself comparatively insoluble; but its salts dissolve easily, and have been found to be of therapeutic value, both locally and internally. We have given it in doses of ten grains three times daily, in a case of phthisis with intestinal ulceration, and it gave rise to no evidence of irritation.

---

**Cream of Malt. Liq. Caulophyllin et Pulsatillæ Co.**—OPPENHEIMER BROS. & Co., London.

The first of these compounds, miscible with water in all proportions, is described as a chemical mixture of malt, cod-liver oil, and hypophosphites, which will be retained by the most delicate stomachs, even when other preparations of malt and cod-liver oil are rejected. Most patients take it well, and some prefer it to all other cod-liver oil mixtures; but there are others who prefer the pure oil, and appear to digest it without difficulty.

The second preparation—described as "the new uterine tonic and restorative"—is said to exert its power principally over the kidneys, bladder, ureters, urethra, uterus, and vagina, and to stimulate the assimilating organs. There is some evidence in favour of its utility; and it has created for itself a very considerable demand in the treatment of irregular, painful, suppressed, and excessive menstruation, a class of chronic disease for which we cannot have too many drugs for alternate or occasional use.

---

**Antiseptic Dressings.**—John Milne, London.

We have received an assorted series of specimens of these well-known and excellent antiseptic dressings.

Surgeon Ulick Bourke's First Field Dressing, consisting of alembroth absorbent cotton-wool, gauze, and bandage, with a little levigated iodoform, seems to be admirably suited for the purpose. The cardboard box in which the dressings are contained adds considerably to the bulk, and would easily get broken.

---

**Glycerine and Codeia Tablets.**—J. Mortimer, Clifton, Bristol.

These convenient tablets contain one-eighth of a grain of codeia in each. They are likely to be useful in coughs of all kinds, more especially in the treatment of phthisis. We have ascertained that—unlike many glycerine jujubes so-called—they do not contain any sugar, and therefore are unobjectionable in cases of diabetes, where all the usual cough syrups are inadmissible. They form an easy and convenient method of administration of codeia in small doses for diabetes, and where the disease is accompanied by chronic lung disease are of the greatest value.

---

**Cascara Sagrada, Hawley** (in Capsules). — Evans, Lescher, & Webb, London.

Mr. J. T. Long, of the Hotwells, Bristol, sends us some of these.

Cascara capsules, by various makers, are now well known, and are much liked by those patients who require a gentle stimulant to peristaltic action. One taken regularly each night, or in some cases two in the day, will usually suffice to counteract chronic constipation. They are easily swallowed, and the nauseous taste of the extract is not perceived. Each capsule is equivalent to half a drachm of the fluid extract of cascara sagrada.

---

**Wood Wool Preparations.**—THE SANITARY WOOD WOOL COMPANY LIMITED, London.

There is no doubt that wood-fibre is, even under great pressure, one of the most absorbent of materials. It is also comparatively cheap. These attributes would make it suitable as a surgical dressing if the individual fibres were longer and the bulk more coherent.

To accomplish the latter object a quantity of ordinary cotton-wool is added, and the mixture forms a soft, elastic and highly absorbent material very suitable for a surgical dressing.

